# Delayed surgery among patients diagnosed with spinal disorders: Retrospective analysis

**DOI:** 10.1371/journal.pone.0325810

**Published:** 2025-06-30

**Authors:** Linda S. Aglio, Tayisha Examond, Samuel A. Justice, Laura Mendez-Pino, Elisabetta Mezzalira, Leah A. Baez, Nicole J. Kelly-Aglio, Kara G. Fields, Robert N. Jamison, Robert R. Edwards, Sarah M. Corey

**Affiliations:** 1 Department of Anesthesiology, Perioperative and Pain Medicine, Brigham and Women’s Hospital, Boston, Massachusetts, United States of America; 2 Department of Neurosurgery, Computational Neurosurgical Outcome Center, Brigham and Women’s Hospital, Boston, Massachusetts, United States of America; 3 Department of Diagnostics and Public Health, University of Verona, Verona, Italy; Duke University Medical Center: Duke University Hospital, UNITED STATES OF AMERICA

## Abstract

To determine the association between race and access to healthcare services with respect to the treatment of spinal cord disorders, a retrospective cohort study of patients receiving an initial diagnosis, two Boston hospitals, September 1, 2017, to June 1, 2018, follow-up through December 31, 2019. Data from patients (18–89 years) diagnosed with spinal cord disorders were extracted retrospectively from a centralized database. Kaplan-Meier curves and multivariable Cox proportional hazards models analyzed the time to spine surgery following initial diagnosis. Patient race was the primary explanatory variable, with five racial groups (Asian, Black, Hispanic, Other, and White) based on a combination of their self-reported race and ethnicity. Hispanic ethnicity (regardless of race), non-Hispanic ethnicity (designated Asian, Black, or White), and “Other” (non-Hispanic patients who designated their race as other than Asian, Black, or White; this included American Indian, Alaska Native, Native Hawaiian or other Pacific Islander, or two or more races). Among 56,186 patients (4% Asian, 7% Black, 5% Hispanic, 6% Other, 77% White) meeting inclusion criteria, Asian (hazard ratio (HR) 0.67 (0.55, 0.82)), Black (HR 0.55 (0.47, 0.63)), Hispanic (HR 0.43 (0.35, 0.52)), and Other (HR 0.59 (0.51, 0.69)) patients had significantly longer times to surgery compared with White patients.

## Introduction

Racial/ethnic disparities in healthcare continue to be a concern in the U.S as marginalized communities often face inequities in terms of access, treatment, outcomes, and overall well-being. Racial and ethnic gaps in the quality of care are likely contributors to health disparities within the healthcare [1]. Individuals from Black, Hispanic, Asian, and other populations frequently face unequal access to crucial medical care. For example, Black patients are less likely to receive cardiac surgery and kidney transplants [2,3]. It is essential to address these racial inequities by equitable distribution of healthcare resources and services, especially in the field of spine cord disorders, which likely contribute to health disparities within the healthcare system.

The field of spine cord disorders is no exception to these disparities. Spine surgery and diagnosis of spinal cord conditions such as herniated disc, spinal stenosis, and degenerative scoliosis are critical for improving the quality of life of affected individuals. The degradation of the spine can significantly impact an individual’s ability to perform daily activities and to function independently due to pain and limited mobility [4,5]. Diagnosis of spinal cord disorders is typically made through a combination of physical examination, imaging studies such as X-rays or MRI [6], and patient history, and early diagnosis is important for improving outcomes; for example, patients who received early acute spinal injury surgery compared to patients who received delayed services for spinal intervention demonstrated a 2-or -more grade improvement [7]. Therefore, early diagnosis allows for a more targeted approach to treatment, which can lead to more effective results and reduced risk of complications. Despite advancements in ongoing acknowledgements of racial disparities in medicine, disparities remain, and surgery is no exception [8]. African American and Black patients are less likely than White patients to undergo certain spinal surgeries or receive diagnostic [9,10]. While there is a significant amount of data available on health disparities among racial/ethnic minorities, there is limited research on timely diagnosis and particularly regarding surgical intervention among these populations. The aim of this study was to determine if racial/ethnic minoritized populations, compared to their White counterparts, differ in terms of time from diagnosis to treatment of spinal cord disorders, including time to spine surgery, physical therapy, injections, and imaging. By focusing on these aspects, our research seeks to provide a more comprehensive understanding of the disparities in the diagnoses and treatment of spinal cord disorders and contribute to the broader effort to achieve equitable healthcare.

## Materials and methods

This study is a retrospective analysis of electronic health record (EHR) data from Mass General Brigham’s (MGB) Research Patient Data Registry (RPDR), a centralized clinical data warehouse collecting data from both current and legacy hospital systems across MGB. The MGB Institutional Review Board approved and waived informed consent. Patients were identified for inclusion in the study through the appearance of a spinal cord disorder diagnosis in their medical record at Brigham and Women’s Hospital (BWH) or Massachusetts General Hospital (MGH) between September 1, 2017 and June 1, 2018; a comprehensive list of International Classification of Diseases Tenth Revision (ICD-10) diagnosis codes used to identify spinal cord disorders is provided in S6 Table in the supplementary material. Patient follow-up visits at the two hospitals were used to identify the study outcomes and were collected through December 31, 2019.

The primary outcome was the time between the date of first spinal cord disorder diagnosis in a patient’s medical record and their date of first spine surgery. Secondary outcomes were time between the date of first spinal cord disorder diagnosis and date of first spine injection, physical therapy session, X-ray, and/or MRI. Diagnoses were identified via appearance of ICD-10 diagnosis codes in a patient’s medical record, while procedures (spine surgery, injection, and physical therapy) were identified via presence of Current Procedural Terminology (CPT) or Healthcare Common Procedure Coding System (HCPCS) codes. Spine physical therapy was specifically identified by the occurrence of both a spine cord disorder diagnosis and a physical therapy procedure in the same patient encounter. Spine X-ray and MRI were identified through patients’ radiology test records. Lists of various codes used to identify the outcomes in the data are provided in S2-4 Tables in the supplementary material.

The data for this research study was accessed and analyzed between July 1, 2022 and July 31, 2024.

### Racial groups

Patient race/ethnicity was the primary explanatory variable, with five racial groups (Asian, Black, Hispanic, other, and White) based on a combination of their self-reported race and ethnicity. As in previous studies [11], patients whose ethnicity was Hispanic were designated as Hispanic regardless of their race/ethnicity, while patients reporting non-Hispanic ethnicity were designated as Asian, Black, or White according to their indicated race/ethnicity. Non-Hispanic patients who designated their race/ethnicity as other than Asian, Black, or White were classified in the “other” racial/ethnic category. These included patients who reported their race/ethnicity as American Indian or Alaska Native, Native Hawaiian or other Pacific Islander, other, or two or more races/ethnicities.

### Spinal cord disorder types

Spinal cord disorder diagnoses were categorized into five mutually exclusive types: deformity, degenerative condition, infection, symptom/syndrome, and trauma. Although a particular spinal cord disorder diagnosis could belong to only one of the preceding five categories, a patient could potentially receive multiple spinal cord disorder diagnoses. Patients were additionally classified as either having an acute or chronic spine condition. Lists of ICD-10 diagnosis codes used to identify the various spinal cord disorder types are provided in S6 Table in the supplementary material.

### Median household income by ZIP code

Median household income in US dollars by ZIP code was obtained for patients via data from the 2018–2022 American Community Survey available through the University [12].

### Statistical analysis

Patient characteristics were summarized and compared between White patients and the four racial/ethnic minoritized patient groups (Asian, Black, Hispanic, and other) using median/interquartile ranges (IQRs) and Wilcoxon rank sum tests for continuous variables and counts/percentages and chi-square tests for categorical variables. All five time-to-event outcomes were compared between the racial groups using both Kaplan-Meier curves and multivariable Cox proportional hazards models adjusted for various spinal cord disorder types (deformity, degenerative condition, infection, symptom/syndrome, and trauma), acute versus chronic spine condition, Medicaid/MassHealth coverage, age, sex, BMI, median household income by ZIP code, and twelve comorbidities comprising the Charlson Comorbidity Index (see S5 Table in the supplementary material for lists of ICD-10 diagnosis codes used to identify these and other [13–15]). A hazard ratio (and 95% confidence interval) below 1 for comparing a particular racial minority patient group to White patients indicated a longer relative time to treatment for the minority group, while a hazard ratio above 1 indicated a shorter relative time to treatment. The proportional hazards assumption was checked via both graphical and analytic examination of the Schoenfeld residuals. Given that there was clear evidence of nonproportional hazards for each of the outcomes, restricted mean survival times (RMSTs) were additionally compared between White patients and each of the four minority patient groups, with truncation at the time of last patient follow-up. All RMST differences were adjusted for the above covariates according to the method of Conner et al. 2019 [16]. In addition to a complete case analysis, multiple imputation for the Cox proportional hazards models was performed as a sensitivity analysis using the R package MICE (with 30 imputations) to account for missing data with respect to patient race/ethnicity and BMI [17].

No references were found in the literature on which to base an *a priori* power calculation for the primary outcome of time to initial spine surgery following first diagnosis of spinal cord disorder. Investigators thus estimated that approximately 30% of patients diagnosed with a spinal cord disorder would have initial spine surgery within 1.5 years of diagnosis. Investigators also determined that a minimum 50% decrease in the hazard of spine surgery associated with being a racial minority as compared to being white would be clinically meaningful. Assuming a racial/ethnic breakdown of 5% Asian, 7.5% Black, 5% Hispanic, 7.5% other, and 75% White patients based on preliminary estimates obtained from MGB’s RPDR Query Tool, a power analysis determined that inclusion of 2,100 patients would provide greater than 90% power at a two-sided alpha level of 0.0125 (0.05/4 pairwise comparisons) to detect a hazard ratio for spine surgery of 0.5 between each of the four minority patient groups and White patients using a Cox proportional hazards model.

All statistical hypothesis tests were two-sided with a p-value < 0.05 indicating statistical significance. Statistical analyses were performed using R software version 4.3.1 (R Foundation for Statistical Computing, Vienna, Austria).

## Results

**Fig 1:**
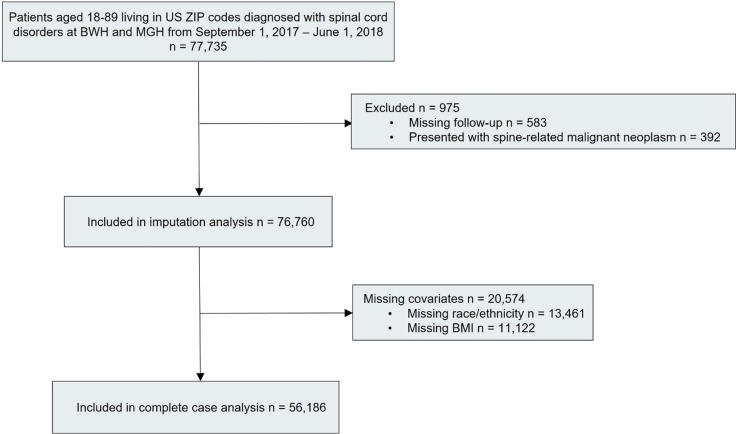
Study flow diagram. Flow diagram outlining how the final study population was selected. We began with 77,735 patients diagnosed with a spinal cord disorder at BWH or MGH between September 2017 and June 2018. After removing those with spine-related malignancies or missing key data (follow-up, race/ethnicity, or BMI), 56,186 patients were included in the final analysis.

There were 77,735 patients aged 18–89 receiving a spinal cord disorder diagnosis at BWH or MGH during the period of September 1, 2017—June 1, 2018. After excluding patients presenting with spine-related malignant neoplasms as well as those patients missing follow-up data, race/ethnicity data, and BMI data, 56,186 patients remained for inclusion in the complete case analysis (Fig 1). Patient characteristics at the time of initial spinal cord disorder diagnosis are presented in Table 1 for the five racial groups. Over three-quarters of included patients were White. Compared to White patients, minority patients were younger, more likely to be female, to be Medicaid/MassHealth beneficiaries, and to have a spinal cord disorder that was a symptom/syndrome. Minority individuals were less likely to have a spinal cord disorder that was a deformity or a degenerative condition and to have a (non-spine-related) malignancy. Patients identifying as Black, Hispanic, and other had higher BMIs than White patients, lived in ZIP codes with lower median household incomes, were more likely to have an acute (as opposed to chronic) spine condition, less likely to have a metastatic solid tumor, and more likely to have AIDS/HIV. These findings were all statistically significant.

**Table 1:** Patient demographics.

Variable	Asian n = 1,984	Black n = 4,212	Hispanic n = 2,967	Other n = 3,582	White n = 43,441
Age (years)	52.92 [38.43, 67.36]*	53.44 [39.05, 64.88]*	49.50 [35.50, 62.08]*	48.97 [36.25, 61.69]*	60.59 [48.17, 71.17]
BMI	24.00 [21.80, 27.00]*	29.00 [25.00, 34.00]*	28.00 [25.00, 32.00]*	28.09 [25.00, 33.00]*	27.00 [23.00, 31.00]
Median Household Income by ZIP Code ($)	137,015.00 [98,813.00, 185,165.00]*	83,913.00 [71,313.00, 121,538.00]*	82,246.00 [66,175.00, 112,750.00]*	84,176.00 [69,620.00, 126,429.00]*	127,679.00 [101,691.00, 162,439.00]
Sex = Male	733 (36.9)*	1,529 (36.3)*	954 (32.2)*	1,220 (34.1)*	18,945 (43.6)
Medicaid/MassHealth Beneficiary	420 (21.2)*	1,792 (42.5)*	1,608 (54.2)*	1,726 (48.2)*	7,007 (16.1)
Spinal Cord Disorders					
Deformity	240 (12.1)*	436 (10.4)*	178 (6.0)*	282 (7.9)*	7,632 (17.6)
Degenerative Condition	262 (13.2)*	413 (9.8)*	197 (6.6)*	310 (8.7)*	7,209 (16.6)
Infection	1 (0.1)	11 (0.3)	4 (0.1)	6 (0.2)	106 (0.2)
Symptom/Syndrome	1,675 (84.4)*	3,695 (87.7)*	2,691 (90.7)*	3,200 (89.3)*	34,264 (78.9)
Trauma	101 (5.1)	197 (4.7)	125 (4.2)*	130 (3.6)*	2,245 (5.2)
Acute	161 (8.1)	1,013 (24.1)*	680 (22.9)*	623 (17.4)*	3,766 (8.7)
Comorbidities from Charlson Comorbidity Index					
Congestive Heart Failure	36 (1.8)*	220 (5.2)*	79 (2.7)*	104 (2.9)*	1,786 (4.1)
Dementia	17 (0.9)	33 (0.8)	25 (0.8)	30 (0.8)	451 (1.0)
Chronic Pulmonary Disease	139 (7.0)*	563 (13.4)*	282 (9.5)	423 (11.8)*	4,182 (9.6)
Rheumatologic Disease	46 (2.3)	139 (3.3)	40 (1.3)*	98 (2.7)	1,220 (2.8)
Mild Liver Disease	107 (5.4)*	151 (3.6)	77 (2.6)*	193 (5.4)*	1,572 (3.6)
Diabetes with Chronic Complications	92 (4.6)*	349 (8.3)*	108 (3.6)	201 (5.6)*	1,594 (3.7)
Hemiplegia/Paraplegia	25 (1.3)	78 (1.9)	26 (0.9)*	40 (1.1)*	728 (1.7)
Comorbidities from Charlson Comorbidity Index					
Renal Disease	75 (3.8)	349 (8.3)*	92 (3.1)*	140 (3.9)*	2,049 (4.7)
Malignancy	204 (10.3)*	309 (7.3)*	136 (4.6)*	205 (5.7)*	5,115 (11.8)
Moderate/Severe Liver Disease	12 (0.6)	7 (0.2)*	5 (0.2)	8 (0.2)	165 (0.4)
Metastatic Solid Tumor	67 (3.4)	73 (1.7)*	32 (1.1)*	51 (1.4)*	1,445 (3.3)
AIDS/HIV	3 (0.2)	74 (1.8)*	16 (0.5)*	37 (1.0)*	135 (0.3)

Categorical variables summarized as count (%); continuous variables summarized as median [IQR]

*P-value < 0.05 for the comparison between the racial minoritized group and White patients; p-values correspond to chi-square tests for categorical variables and Wilcoxon rank sum tests for continuous variables

**Fig 2:**
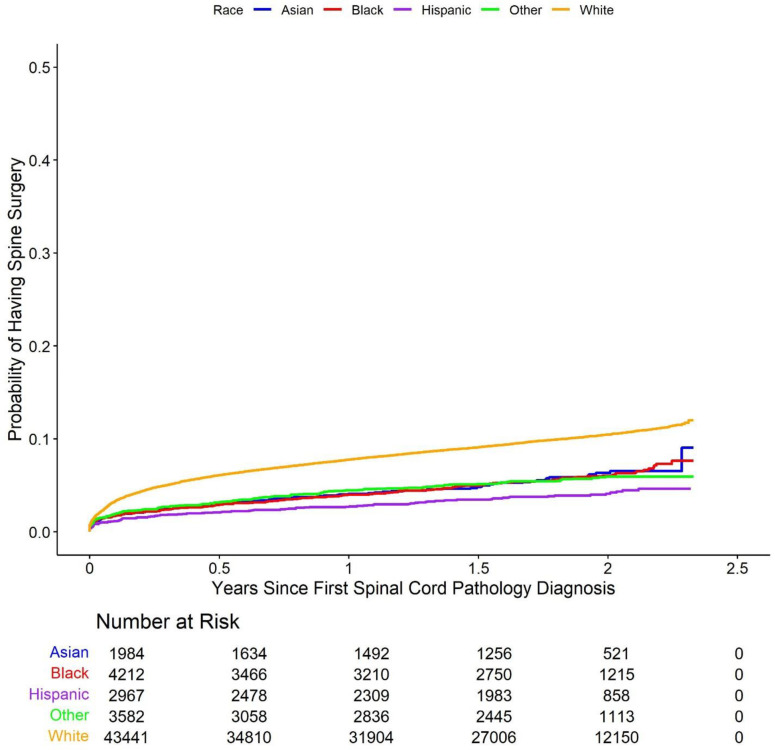
Cumulative incidence of Kaplan-Meier curve plots comparing outcomes between the racial groups. These Kaplan-Meier curves show the cumulative probability of receiving (2) spine surgery, (3) spine injection, (4) spine physical therapy, (5) spine X-ray, and (6) spine MRI within after an initial diagnosis of spinal cord pathology, broken down by racial group. Across these types of care, White patients demonstrated the highest cumulative incidence of surgery compared to other groups across the study period. Racial disparities are evident, with Asian, Black, Hispanic, and Other racial groups showing consistently lower probabilities of surgery.

**Fig 3:**
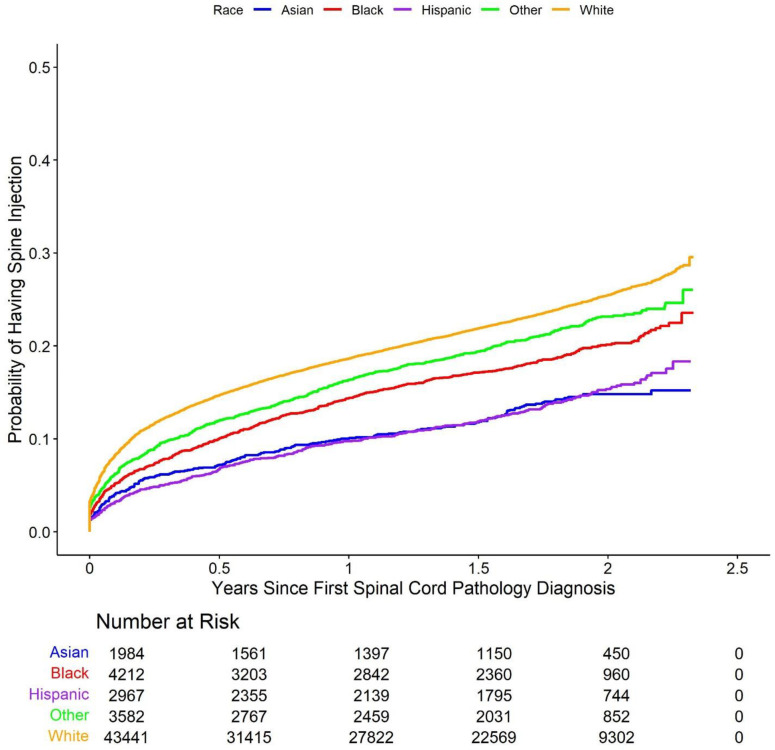
Cumulative incidence of Kaplan-Meier curve plots comparing outcomes between the racial groups. Cumulative incidence of receiving a spine injection after diagnosis, by racial group. Minority patients were less likely than White patients to receive injections over time, highlighting disparities in care access.

**Fig 4:**
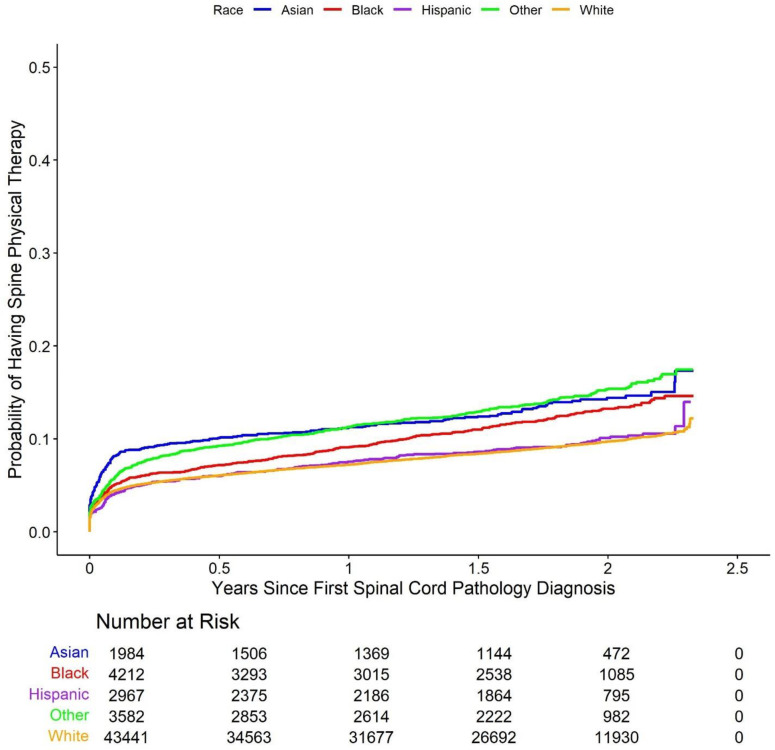
Cumulative incidence of Kaplan-Meier curve plots comparing outcomes between the racial groups. Kaplan-Meier cumulative incidence curves comparing the probability of receiving spine physical therapy across racial groups. The analysis shows that Asian, Black, and patients categorized as Other were more likely to receive spine physical therapy over time compared to White patients.

**Fig 5:**
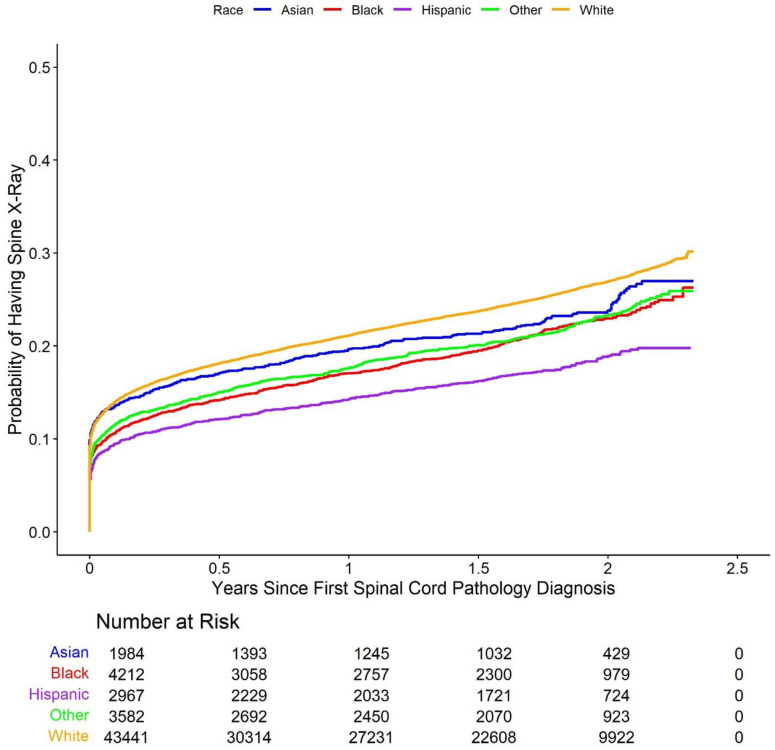
Cumulative incidence of Kaplan-Meier curve plots comparing outcomes between the racial groups. Rates of spine X-ray utilization over time after diagnosis, shown across racial groups. White patients had higher imaging rates over time compared to minority groups.

**Fig 6:**
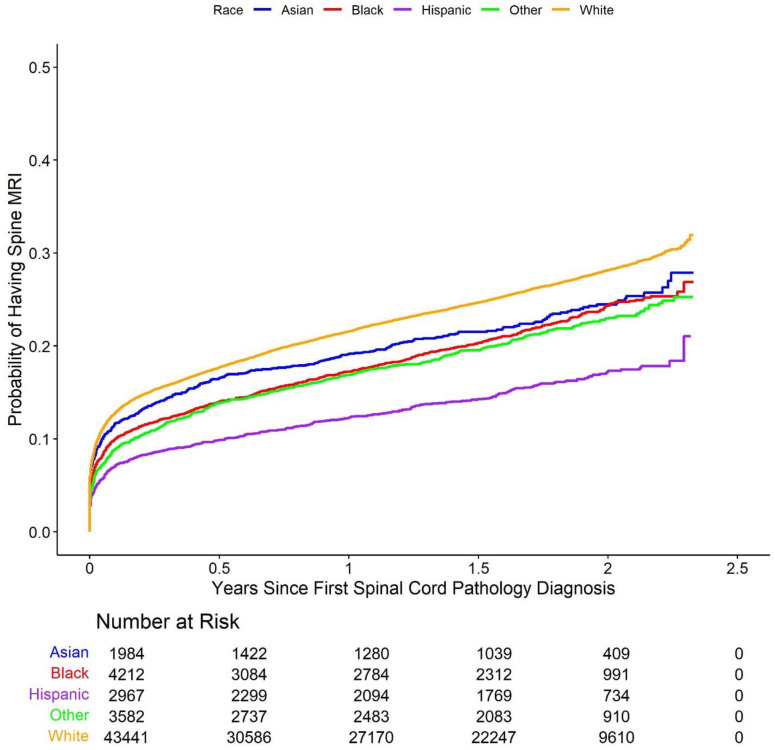
Cumulative incidence of Kaplan-Meier curve plots comparing outcomes between the racial groups. Cumulative probability of undergoing spine MRI following initial diagnosis, stratified by racial group. As with surgery and X-rays, White patients were more likely to receive MRI over the study period, with lower cumulative incidence observed among Asian, Black, Hispanic, and Other racial groups.

Cumulative incidence Kaplan-Meier curve plots comparing the five racial groups with respect to each of the study outcomes are shown in Figs 2–6.

These plots suggest that minority patients were generally less likely than White patients to receive spine surgery, injection, X-ray, and MRI over the duration of the study following initial spinal cord disorder diagnosis. On the other hand, Asian, Black, and other patients appear more likely than White patients to have received spine physical therapy. Multivariable Cox proportional hazards models largely confirmed these results. The model for the primary outcome demonstrated that Asian (hazard ratio (HR) 0.67, 95% confidence interval (CI) (0.55, 0.82), p-value < 0.001), Black (HR 0.55 (0.47, 0.63), p-value < 0.001), Hispanic (HR 0.43 (0.35, 0.52), p-value < 0.001), and other (HR 0.59 (0.51, 0.69), p-value < 0.001) patients all had longer times to surgery than White patients (note again that hazard ratios below 1 indicate a longer relative time to treatment for the racial minority patient groups compared to White patients, while hazard ratios above 1 indicate a shorter relative time to treatment). Results obtained via multiple imputation were similar and are summarized in S1 Table in the supplementary material. The results of the Cox proportional hazards models were further corroborated via comparison of restricted mean survival times between White patients and each of the four minority patient groups (Table 2). The (unadjusted) average time to initial spine surgery over the study period was 814 days for Asian patients (adjusted RMST difference compared to White patients 24 days, 95% CI (15, 33), p-value < 0.001), 814 days for Black patients (RMST difference 31 (25, 38) days, p-value < 0.001), 822 days for Hispanic patients (RMST difference 27 (19, 34) days, p-value < 0.001), and 814 days for other patients (RMST difference 17 (9, 24) days, p-value < 0.001), and 784 days for White patients.

**Table 2:** Summary of model results for outcomes.

Outcome	Race	Adjusted HR (95% CI)	P-Value	Unadjusted RMST (Days)	Adjusted RMST Difference (95% CI)	P-Value
Time to Spine Surgery						
	White	---	---	784	---	---
	Asian	0.67 (0.55, 0.82)	<0.001	814	24 (15, 33)	<0.001
	Black	0.55 (0.47, 0.63)	<0.001	814	31 (25, 38)	<0.001
	Hispanic	0.43 (0.35, 0.52)	<0.001	822	27 (19, 34)	<0.001
	Other	0.59 (0.51, 0.69)	<0.001	814	17 (9, 24)	<0.001
Time to Spine Injection						
	White	---	---	688	---	---
	Asian	0.58 (0.51, 0.66)	<0.001	759	67 (55, 79)	<0.001
	Black	0.75 (0.70, 0.82)	<0.001	727	39 (27, 50)	<0.001
	Hispanic	0.57 (0.51, 0.64)	<0.001	760	65 (52, 77)	<0.001
	Other	0.91 (0.84, 0.99)	0.027	708	15 (1, 28)	0.033
Time to Spine Physical Therapy						
	White	---	---	787	---	---
	Asian	1.45 (1.27, 1.65)	<0.001	751	−33 (−46, −20)	<0.001
	Black	1.23 (1.11, 1.36)	<0.001	768	−16 (−26, −7)	0.001
	Hispanic	0.93 (0.81, 1.06)	0.253	782	−9 (−21, 2)	0.113
	Other	1.44 (1.30, 1.59)	<0.001	751	−28 (−40, −17)	<0.001
Time to Spine X-Ray						
	White	---	---	665	---	---
	Asian	1.02 (0.93, 1.13)	0.636	680	−5 (−21, 11)	0.539
	Black	0.94 (0.87, 1.01)	0.089	699	22 (8, 35)	0.002
	Hispanic	0.81 (0.74, 0.89)	<0.001	721	41 (26, 55)	<0.001
	Other	1.00 (0.92, 1.08)	0.917	694	1 (−14, 16)	0.913
Time to Spine MRI						
	White	---	---	662	---	---
	Asian	0.91 (0.82, 1.00)	0.049	684	13 (−3, 29)	0.119
	Black	0.86 (0.80, 0.93)	<0.001	697	24 (10, 37)	<0.001
	Hispanic	0.62 (0.56, 0.69)	<0.001	738	71 (56, 85)	<0.001
	Other	0.84 (0.78, 0.91)	<0.001	702	23 (9, 38)	0.001

HR: Hazard Ratio; RMST: Restricted Mean Survival Time

As with the Kaplan-Meier curves, models for the outcome of time to initial spine physical therapy session demonstrated qualitatively different results than the models for the other four outcomes. Asian (HR 1.45 (1.27, 1.65), p-value < 0.001), Black (HR 1.23 (1.11, 1.36), p-value < 0.001), and other (HR 1.44 (1.30, 1.59), p-value < 0.001) patients all had shorter times to physical therapy than White patients according to the Cox proportional hazards model, while there was no difference in time to physical therapy between Hispanic (HR 0.93 (0.81, 1.06), p-value = 0.253) and White patients. Furthermore, the average time to initial spine physical therapy session over the study period was 751 days for Asian patients (RMST difference −33 (−46, −20) days, p-value < 0.001), 768 days for Black patients (RMST difference −16 (−26, −7) days, p-value = 0.001), 782 days for Hispanic patients (RMST difference −9 (−21, 2) days, p-value = 0.113), and 751 days for other patients (RMST difference −28 (−40, −17) days, p-value < 0.001), and 787 days for White patients.

## Discussion

The present study examined disparities in access to spine-related healthcare services among 56,186 patients diagnosed with spinal cord disorders. Our findings indicated that minority patients (encompassing Asian, Black, Hispanic, and other individuals) experienced significantly longer waiting periods prior to surgery. In addition, we identified significant gaps in access to other essential spinal services, including spine injections, MRI, and X-rays. Interestingly, the timing of physical therapy interventions to spinal disorders showed a different trend, with White patients experiencing delays in accessing this form of care compared to minority patients. Collectively, the delayed time to surgery for minority patients not only prolongs their suffering from spinal conditions but also underscores the persistence of inequalities within the healthcare system leading to inferior health outcomes.

Minority patients face formidable barriers to receiving timely surgical interventions [18], which may contribute to the previously established and notable gap in race/ethnicity -related outcomes following the diagnosis of spinal disorder. These potential barriers, including financial constraints [19–21], limited healthcare access [22], healthcare provider bias [23], and socioeconomic disparities [24] contribute to the established gap in race/ethnic -related outcomes subsequent to the diagnosis of spinal disorder. Researchers have identified a pressing need for further research on race/ethnic-specific outcomes following the diagnosis of [25,26]. These studies and reviews have demonstrated that minority patients experience delays in accessing surgical interventions. Particularly noteworthy is the alarming 43% higher likelihood of delayed surgery observed among African Americans [27].

Additionally, our study findings align with previous research on racial/ethnic disparities in access to surgical intervention. A recent study conducted by Massaad [28] demonstrated similar results in a smaller sample of patients undergoing lumbar spinal stenosis. Within the broader context of healthcare disparities, a delay in accessing spine injections poses a significant challenge, particularly among minority populations. Prolonged pain and decreased functionality may lead to additional healthcare utilization, including increased visits to primary care providers, (as patients seek alternative forms of relief for their spinal conditions) or potentially increased pain medication usage. Over the decades, epidural steroid injection (ESI) in the management of both low back pain and sciatica, have offered patients relief from symptoms and improved overall quality of life [29]. In considering broader healthcare disparities, it becomes apparent that delayed access to spine injections poses a significant challenge, particularly among minority populations. Furthermore, discrepancies in the timing of spine X-rays and MRIs compound these challenges, heightening inequities in timely and comprehensive spinal care.

Ensuring timely access to X-rays and MRIs is crucial for effective management and diagnosis of spinal disorders. MRI has emerged as a vital method for imaging neurological tissues, including the spinal cord, setting the benchmark for diagnostic imaging and guiding clinical decision making [30]. However, our study revealed disparities in the timing of these diagnostic procedures, further widening healthcare inequities among minority populations. The study displays that minority patients, including Black, Hispanic, Asian, and other individuals, faced extended waiting periods for spine X-rays and MRIs compared to their White counterparts. Black patients have substantially lower odds of receiving spinal imaging [10], a finding we replicate here in the form of delays in access to spinal MRI among patients who are members of racial minority groups relative to white patients. This delay not only impedes prompt diagnosis and treatment initiation but also prolongs patients’ distress and compromises their overall well-being. Moreover, delayed access to diagnostic imaging may contribute to the progression of spinal conditions, necessitating more invasive and costly interventions in the long term. With the increasing prevalence of spinal disorders [31], recent research indicates a significant surge in lumbar MRI utilization among Medicare beneficiaries, with rates escalating by up to 300 percent between 1994 and 2006 [32]. Thus, addressing these disparities in care and ensuring equitable access to timely diagnostic procedures are essential steps in enhancing healthcare outcomes for all patient demographics.

Disparities in referrals clearly contribute to this pattern of findings; a 2021 study showed a decreased rate and speed of Black patients being referred to specialists [33], highlighting the necessity of analyzing possible obstacles in the referral process, guaranteeing equal opportunities for timely surgical evaluation regardless of race/ethnicity. Institutions like MGB treat patients in the ER regardless of insurance status, but for other services, having insurance contracted with MGB is crucial. Those with the appropriate insurance are referred by their primary care physician (PCP) or specialist, with their insurance covering the costs. Without compatible insurance, patients may face higher out-of-pocket costs or may not be treated at all. The referral process involves an initial consultation, insurance verification, referral submission, appointment scheduling, and follow-up care. For specialties, this ensures patients receive expert care from MGB’s medical teams, with insurance compatibility being essential for access. Primary care physicians (PCPs) and community clinics play a crucial role as gatekeepers of healthcare access. They must be integral parts of the referral network to mitigate disparities. To address healthcare disparities, it is essential to implement culturally appropriate interventions that tackle healthcare literacy, language barriers, and cultural and religious norms. Establishing a therapeutic alliance is crucial for building trust with these patients. These upstream drivers significantly impact healthcare access, and the pandemic has only deepened the inequalities in these communities. Interestingly, in the present study we found white patients experienced delays in time to spine physical therapy, a finding that contrasts with some prior research [34], though at least one study noted that Black and Hispanic patients were more likely to access physical therapy services for pain compared to White patients who were more likely to receive back surgery [35]. In addition, prior studies have consistently demonstrated that Black and Hispanic patients are less likely to attend physical therapy sessions compared to their White counterparts [34,36,37]. These disparities may be influenced by various barriers such as financial resources, transportation challenges, and inadequate health insurance coverage which hinder access to physical therapy. As minority patients are less likely to receive specialist care compared to their White counterparts, this reveals significant disparities in access to specialized healthcare services [33].

Racial and ethnic minoritized experience greater bias and discrimination, which has been associated with more intense pain symptoms [38]. Fifty percent of Hispanic and 70% of Black individuals in the United States have experienced discrimination or have been treated [38]. Perceived racial discrimination was associated with elevated joint pain intensity among African American women with a diagnosis of [39,40]. It was also confirmed as the strongest predictor of back pain among African American patients [41]. Perceived discrimination’s association with more bodily pain has also been reported among Chinese individuals [42,43]. In addition to the impact on psychological well-being, perceived discrimination is also linked to increased psychological stress, anxiety, and other negative affective factors, which can exacerbate the experience of pain. Studies consistently show that racial and ethnic minoritized patients report experiencing discrimination in their interactions with healthcare providers, influencing both the assessment and treatment plans for pain management. Hirsh and colleagues found that race/ethnicity influences both the assessment and treatment plans for individuals with pain [44,45].

Hispanic patients often report mistrusting their healthcare providers as they seek a patient-centered approach to their care [46,47]. Communication between patients and providers is essential to shared decision-making and effective health care management [48–50]. Among racial and ethnic minoritized, language barriers, cultural differences, and health care literacy, may impact the patient-provider communication and relationship. There is evidence that educational deficiencies in the medical school training curriculum may contribute to later racial and ethnic biases in the provision of healthcare, with medical students frequently endorsing false beliefs about biological difference between Black and White individuals [51]. Such beliefs appear to be potentially quite pervasive- Injured Black football players as compared to white players were more likely to play in a subsequent game, potentially because it was assumed on the part of decision-makers that Black players experienced less pain. Such disparities in pain-related assumptions and management have likely led to the finding of large racial disparities in pain among retired NFL players, with Black former players reporting more intense pain and more pain-related limitations relative to white former players [52].

There are several limitations of this study that deserve mention. First, we must acknowledge the data limitations inherent in our analysis. Specifically, because we are only examining data from BWH and MGH, we may be missing diagnoses and treatments that occurred at other institutions. Additionally, we might not capture patients’ initial spinal disorder diagnoses at BWH or MGH if these diagnoses occurred before the study period. This means that some diagnoses within our study period may not be the patients’ first, potentially impacting our findings. As a result, our findings may not be generalizable to patients treated in different hospital systems, particularly those in community hospitals, rural settings, or under-resourced healthcare facilities. This limitation highlights the need for a more comprehensive, multi-institutional approach to studying healthcare disparities, particularly in spinal disorder treatment. A broader dataset incorporating a wider range of healthcare settings would provide a clearer and more representative picture of systemic inequities. Beyond the scope of this specific study, addressing these disparities requires systemic reforms aimed at improving healthcare access, reducing barriers to timely diagnosis, and ensuring continuity of care across different institutions. Another limitation of this study is the reliance on historical data, which means that individuals diagnosed earlier in the study period may not be fully represented in the years that follow. This gap can affect our ability to capture the complete treatment journey and outcomes for those patients, as their initial diagnosis and subsequent care might not be recorded in the same detail as for those diagnosed more recently. We used a retrospective design from a multicenter database, limiting the establishment of causal relationships. Also, differing protocols that are standard for participating hospitals to address spinal comorbidities can impact generalizability. It is also essential to acknowledge that our findings may not capture the full spectrum of healthcare experiences and disparities. This sample, which was collected from a specific geographical location, may not fully represent the experiences of minority and White patients in different regions. Furthermore, reliance on medical records introduces the issue of incomplete data and limits the ability to capture real-time variations. There are several reasons that surgery is pursued for treatment of spinal disorder and this information was not available for this study. Finally, we were not able to measure a number of external factors such as cultural competence and cultural background of the providers that could contribute to variations in the observed outcomes. Addressing these limitations in future research will contribute to a comprehensive understanding of health disparities in spinal disorder care.

## Conclusion

These results indicate a significant disparity in the time to imaging and surgery between minoritized and White patients following initial spinal diagnosis. Addressing disparities is critical from an equity standpoint and improving the nation’s health and economic prosperity [53]. To reduce these gaps, we can implement several initiatives. Our analyses suggest that increasing awareness of racial/ethnic healthcare disparities among both the public and healthcare providers is essential, as educational initiatives can help to identify and mitigate biases in treatment decisions. Specifically, healthcare policy should focus on: 1) Raising public and provider awareness of racial/ethnic healthcare disparities, 2) Expanding health insurance coverage, 3) Improving the capacity and number of providers in under-served communities, and 4) Increasing the fund of knowledge on the causes and interventions required to reduce disparities [54]. The importance of community-based awareness and educational interventions is demonstrated in various successful public health efforts. Initiatives that leverage trusted community spaces, such as barber shops and beauty salons [55], have effectively addressed health issues within marginalized communities by providing culturally relevant settings for health education. Similarly, other community-centered programs have successfully increased health screenings and reduced disparities by involving local leaders and healthcare providers [56,57]. These strategies underscore the effectiveness of using culturally aligned and community-driven approaches to raise awareness and encourage equitable healthcare practices, especially when addressing delays in imaging and surgery for minoritized patients. Additionally, our analysis highlights gaps in the current understanding of the causes of these disparities and effective interventions. Increasing research funding to further investigate causes and effective interventions will be vital in reducing health disparities and improving overall outcomes. Concrete progress in these areas can potentially reduce health disparities and improve overall health in the United States.

## Supporting information

S1 Table Summary of multiply imputed Cox proportional hazards model results for outcomes. (PDF)

S2 Table List of CPT codes used to identify spine surgery. (PDF)

S3 Table List of codes used to identify spine injection. (PDF)

S4 Table List of codes used to identify spine physical therapy session; for a patient to be considered to have spine physical therapy, he/she had to be flagged in the same encounter for both one of the CPT or HCPCS codes and one of the ICD-10 diagnosis codes. (PDF)

S5 Table List of ICD-10 diagnosis codes used to identify various comorbidities. (PDF)

S6. Table Comprehensive reference table providing lists of ICD-10 diagnosis codes grouped into categories for spine and musculoskeletal conditions. (PDF)
