# Supplementary material for: Delayed surgery among patients diagnosed with spinal disorders: Retrospective analysis
Source: PLoS One. 2025 Jun 30;20(6):e0325810. doi: 10.1371/journal.pone.0325810 (PMC12208456; doi:10.1371/journal.pone.0325810)
Supplement: S1 Table — (PDF) [file pone.0325810.s001.pdf]

S1 Table. Summary of multiply imputed Cox proportional hazards model results for outcomes.

Outcome	Race	Adjusted HR (95% CI)	P-Value
**Time to Spine Surgery**			
	White	---	---
	Asian	0.65 (0.54, 0.79)	<0.001
	Black	0.56 (0.49, 0.64)	<0.001
	Hispanic	0.43 (0.36, 0.53)	<0.001
	Other	0.61 (0.53, 0.70)	<0.001
**Time to Spine Injection**			
	White	---	---
	Asian	0.60 (0.54, 0.68)	<0.001
	Black	0.75 (0.70, 0.81)	<0.001
	Hispanic	0.59 (0.53, 0.66)	<0.001
	Other	0.89 (0.83, 0.96)	0.003
**Time to Spine Physical Therapy**			
	White	---	---
	Asian	1.46 (1.30, 1.65)	<0.001
	Black	1.25 (1.13, 1.37)	<0.001
	Hispanic	0.97 (0.85, 1.10)	0.646
	Other	1.45 (1.32, 1.59)	<0.001
**Time to Spine X-Ray**			
	White	---	---
	Asian	1.07 (0.98, 1.17)	0.152
	Black	0.98 (0.92, 1.05)	0.639
**Outcome**	**Race**	**Adjusted HR (95% CI)**	**P-Value**

**Time to Spine X-Ray**			
	Hispanic	0.86 (0.78, 0.94)	0.001
	Other	1.03 (0.96, 1.10)	0.448
**Time to Spine MRI**			
	White	---	---
	Asian	0.94 (0.86, 1.03)	0.162
	Black	0.88 (0.82, 0.94)	<0.001
	Hispanic	0.66 (0.60, 0.73)	<0.001
	Other	0.87 (0.81, 0.93)	<0.001

HR: Hazard Ratio
