# Supplementary material for: Delayed surgery among patients diagnosed with spinal disorders: Retrospective analysis
Source: PLoS One. 2025 Jun 30;20(6):e0325810. doi: 10.1371/journal.pone.0325810 (PMC12208456; doi:10.1371/journal.pone.0325810)
Supplement: S2 Table — (PDF) [file pone.0325810.s002.pdf]

S2 Table. List of CPT codes used to identify spine surgery.

0219T	22100	22327	22585	22844	27280	63015	63078	63272	63661
0222T	22101	22328	22590	22845	27299	63016	63081	63275	63662
10140	22102	22510	22595	22846	27519	63017	63082	63276	63685
10180	22103	22511	22600	22847	32503	63020	63085	63277	63688
11042	22110	22512	22610	22848	32504	63030	63086	63278	63707
11400	22112	22513	22612	22849	38220	63035	63087	63280	63709
12020	22114	22514	22614	22850	61215	63040	63088	63281	63710
20225	22116	22515	22630	22851	61343	63042	63090	63282	63740
20250	22206	22520	22632	22852	61595	63043	63091	63283	63741
20251	22207	22521	22633	22853	61618	63044	63101	63285	64713
20525	22208	22522	22634	22854	61783	63045	63102	63286	64721
20661	22210	22523	22800	22855	62201	63046	63103	63287	69990
20930	22212	22524	22802	22856	62270	63047	63185	63290	76012
20931	22214	22525	22804	22858	62272	63048	63200	63300	76013
20936	22216	22532	22808	22859	62287	63051	63250	63301	95970
20937	22224	22533	22810	22861	62350	63055	63251	63302	95971
20938	22226	22534	22812	22864	62351	63056	63252	63303	95972
20939	22310	22548	22818	22867	62355	63057	63265	63304	95973
20982	22315	22551	22830	22868	63001	63064	63266	63305	97605
20985	22318	22552	22840	22899	63003	63066	63267	63306	97606
21501	22319	22554	22841	27080	63005	63075	63268	63307	
22010	22325	22556	22842	27218	63011	63076	63270	63308	
22015	22326	22558	22843	27279	63012	63077	63271	63655	
