# Supplementary material for: Delayed surgery among patients diagnosed with spinal disorders: Retrospective analysis
Source: PLoS One. 2025 Jun 30;20(6):e0325810. doi: 10.1371/journal.pone.0325810 (PMC12208456; doi:10.1371/journal.pone.0325810)
Supplement: S3 Table — (PDF) [file pone.0325810.s003.pdf]

S3 Table. List of codes used to identify spine injection.

**CPT Codes**	0275T	22513	62327	64415	64462	64555	64640	72270
	20550	22514	62355	64416	64479	64561	64642	72275
	20551	22515	62362	64417	64480	64575	64643	72285
	20552	22869	62365	64420	64483	64581	64644	72295
	20553	22870	62367	64421	64484	64585	64645	76999
	20600	27096	62368	64445	64486	64590	64646	77003
	20604	36475	62369	64446	64488	64595	64647	95873
	20605	36476	62370	64447	64490	64616	64802	95874
	20606	62273	63650	64448	64491	64624	64804	95970
	20610	62321	63661	64449	64492	64625	64809	95971
	20611	62323	63663	64450	64493	64633	64818	95972
	22510	62324	63685	64451	64494	64634	64999	95990
	22511	62325	63688	64455	64495	64635	65420	95991
	22512	62326	64408	64461	64510	64636	72265	
**HCPCS Codes**	C1778	C1897						
