# Supplementary material for: Delayed surgery among patients diagnosed with spinal disorders: Retrospective analysis
Source: PLoS One. 2025 Jun 30;20(6):e0325810. doi: 10.1371/journal.pone.0325810 (PMC12208456; doi:10.1371/journal.pone.0325810)
Supplement: S4 Table — (PDF) [file pone.0325810.s004.pdf]

S4 Table. List of codes used to identify spine physical therapy session; for a patient to be considered to have spine physical therapy, he/she had to be flagged in the same encounter for both one of the CPT or HCPCS codes and one of the ICD-10 diagnosis codes.

**CPT Codes**	97010	97112	97150	97164
	97012	97113	97161	97530
	97014	97116	97162	97542
	97110	97140	97163	
**HCPCS Codes**	G0283			
**ICD-10 Diagnosis Codes**	M53.3	M54.2	M54.5	M54.6
