# Supplementary material for: Delayed surgery among patients diagnosed with spinal disorders: Retrospective analysis
Source: PLoS One. 2025 Jun 30;20(6):e0325810. doi: 10.1371/journal.pone.0325810 (PMC12208456; doi:10.1371/journal.pone.0325810)
Supplement: S5 Table — (PDF) [file pone.0325810.s005.pdf]

S5 Table. List of ICD-10 diagnosis codes used to identify various comorbidities.

**Congestive Heart Failure**	I09.9, I11.0, I13.0, I13.2, I25.5, I42.0, I42.5, I42.6, I42.7, I42.8, I42.9, I43, I50.x, P29.0
**Dementia**	F01.x, F02.x, F03.x, G30.x, G31.1
**Chronic Pulmonary Disease**	I27.8x, I27.9, J40, J41.x, J42, J43.x, J44.x, J45.x, J47.x, J60, J61, J62.x, J63.x, J64, J65, J66.x, J67.x, J68.4, J70.1, J70.3
**Rheumatologic Disease**	M05.x, M06.x, M31.5, M32.x, M33.x, M34.x, M35.1, M35.3, M36.0
**Mild Liver Disease**	B18.x, K70.0, K70.1x, K70.2, K70.3x, K70.9, K71.3, K71.4, K71.5x, K71.7, K73.x, K74.x, K76.0, K76.2, K76.3, K76.4, K76.8x, K76.9, Z94.4
**Diabetes with Chronic Complications**	E10.2x, E10.3x, E10.4x, E10.5x, E11.2x, E11.3x, E11.4x, E11.5x, E13.2x, E13.3x, E13.4x, E13.5x
**Hemiplegia/Paraplegia**	G04.1, G11.4, G80.1, G80.2, G81.x, G82.x, G83.0, G83.1x, G83.2x, G83.3x, G83.4, G83.9
**Renal Disease**	I12.0, I13.1x, N03.2, N03.3, N03.4, N03.5, N03.6, N03.7, N05.2, N05.3, N05.4, N05.5, N05.6, N05.7, N18.x, N19, N25.0, Z49.0x, Z94.0, Z99.2
**Spine-Related Malignancy**	C41.0, C41.1, C41.2, C41.4, C41.9, C72.x
**Non-Spine-Related Malignancy**	C00.x, C01, C02.x, C03.x, C04.x, C05.x, C06.x, C07, C08.x, C09.x, C10.x, C11.x, C12, C13.x, C14.x, C15.x, C16.x, C17.x, C18.x, C19, C20, C21.x, C22.x, C23, C24.x, C25.x, C26.x, C30.x, C31.x, C32.x, C33, C34.x, C37, C38.x, C39.x, C40.x, C41.3, C43.x, C45.x, C46.x, C47.x, C48.x, C49.x, C50.x, C51.x, C52, C53.x, C54.x, C55, C56.x, C57.x, C58, C60.x, C61, C62.x, C63.x, C64.x, C65.x, C66.x, C67.x, C68.x, C69.x, C70.x, C71.x, C73, C74.x, C75.x, C76.x, C81.x, C82.x, C83.x, C84.x, C85.x, C88.x, C90.x, C91.x, C92.x, C93.x, C94.x, C95.x, C96.x
**Moderate/Severe Liver Disease**	I85.0x, I86.4, K70.4x, K71.1x, K72.1x, K72.9x, K76.5, K76.6, K76.7
**Metastatic Solid Tumor**	C77.x, C78.x, C79.x, C80.x
**AIDS/HIV**	B20
**Motor/Sensory Deficits or Incontinence**	F44.6, F82, G60.9, G90.09, K59.2, M79.2, N39.46, R15.9, R26.9, R29.818, R32, Z74.01, Z74.09
