# Supplementary material for: Delayed surgery among patients diagnosed with spinal disorders: Retrospective analysis
Source: PLoS One. 2025 Jun 30;20(6):e0325810. doi: 10.1371/journal.pone.0325810 (PMC12208456; doi:10.1371/journal.pone.0325810)
Supplement: S6. Table — (PDF) [file pone.0325810.s006.pdf]

S6 Table. Comprehensive reference table providing lists of ICD-10 diagnosis codes grouped into categories for spine and musculoskeletal conditions.

Conditions	ICD-10 Codes
**Deformities**	M40, M40.3, M40.36, M40.37, M40.30, M40.35, M40.5, M40.56, M40.57, M40.50, M40.55, M40.2, M40.29, M40.292, M40.293, M40.299, M40.294, M40.295, M40.20, M40.202, M40.203, M40.209, M40.204, M40.205, M40.1, M40.12, M40.13, M40.10, M40.14, M40.15, M40.0, M40.03, M40.00, M40.04, M40.05, M40.4, M40.46, M40.47, M40.40., M40.45, M41, M41.0, M41.02, M41.03, M41.06, M41.07, M41.08, M41.00, M41.04, M41.05, M41.1, M41.12, M41.122, M41.123, M41.126, M41.127, M41.129, M41.124, M41.125, M41.11, M41.112, M41.113, M41.116, M41. 117, M41.119, M41.114, M41.115, M41.4, M41.42, M41.43, M41.46, M41.47, M41.41, M41.40, M41.44, M41.45, M41.8, M41.82, M41.83, M41.86, M41.87, M41.80, M41.84, M41.85, M41.20, M41.22, M41.23, M41. 26, M41.27, M41.20, M41.24, M41.25, M41.26, M41.50, M41.5, M41.52, M41.53M41. 56, M41. 50, M41.54, M41. 55, M41. 9, M41. 3, M41.30, M41.34, M41.35, M43, M43.9, M43.20, M43.2, M43.22, M43.23, M43.26, M43.27, M43.21, M43.28, M43. 20, M43.24, M43.25, M43.4, M43.5, M43.5X, M43.5X2, M43.5X3, M43.5x, M43.8, M43.8x, M43.3, M43.1, M43.12, M43.13, M43.16, M43.17, M43.11, M43.18, M43.10, M43.14, M43.19, M43.0, M43.02, M43.03, M43.06, M43.07, M43.09, M43.01, M43.08, M43.00, M43.04, M43.05, M43.6, M42, M42.1, M42.12, M42.13, M42.16, M42.17,M42.19, M42.21, M42.18,M42.10,M42.14, M42.15, M42.0, M42.02,M42.03, M42.06, M42.07, M42.09, M42.01, M42.08, M42.00, M42.04, M42.05, M42.9<br><br>M48, M48.1, M48.12, M48.13, M48.16, M48.17, M48.19.11, M48.18, M48.10, M48.14, M48.15, M48.5, M48.52, M48. M48.53, M48.53x, M48.56, M48.56x, M48.57, M48.57x, M48.51, M48.51x, M48.58, M48.58x, M48.50, M48.50x, M48.54, M48.54x, M48.55, M48.55x, M48.4, M48.42, M48.42x, M48.43x, M48.46, M48.46x, M48.47, M48.47x, M48.47XG, M48.40, M48.420x , M48.41, M48.41x, M48.44, M48.44x, M48.45, M48.45XA, M48.59, M48.5x, M48.2, M48.22, M48.23, M48.26, M48.27, M48.21, M48.20, M48.24, M48.25, M48.8, M48.8x, M48.0, M48.02, M48.03, M48.06, M48.07, M48.01, M48.08, M48.00, M48.04, M48.05, M48.9, M48.3, M48.32, M48.33, M48.36, M48.37, M48.31, M48.38, M48.30, M48.34, M48.35<br><br>Q67, Q67.5, Q67.1, Q67.0, Q67.2, Q67.8, Q67.4, Q67.7, Q67.6, Q67.3, Q76, Q76.0, Q76.1, Q76.5, Q76,9, Q76.7, Q76.3, Q76.2, Q76.2, Q76.8, Q76.6, Q76.4, Q76.41, Q76.412, Q76.412, Q76.413, Q76.411, Q76.414, Q76.415, Q76.419, Q76.42, Q76.426, Q76.427, Q76.428, Q76.425, Q76.429 Q76.49
**Degenerative Condition**	M46, M46.4, M46.42, M46.43, M46.46, M46.47, M46.49, M46.41, M46.48, M46.40, M46.44, M46.45, M46.3, M46.32, M46.33, M46.36, M46.37, M46.39, M46.31, M46.38, M46.30, M46.34, M46.35, M46.5, M46.52, M46.53, M46.56, M46.57, M46.59, M46.51, M46.58, M46.50, M46.54, M46.55, M46.8, M46.82, M46.83, M46.86, M46.87, M46.89, M46.81,V.88 M46.80, M46.84, M46.85, M46.1, M46.81, M46.88, M46.80, M46.84, M46.85, M46.0, M46.02, M46.03, M46.06, M46.07, M46.01, M46.08, M46.00, M46.04, M46.05, M46.9, M46.92, M46.93, M46.96, M46.97, M46.99, M46,91, M46.98, M46.90, M46.94, M46.95, M47, M47.0, M47.012, M47.013, M47.016, M47.011, M47.019, M47.014, M47.015, M47.022, M47.021, M47.029, M47.8, M47.89, M47.892, M47.893, M47.896, M47.897, M47.891, M47.892, M47.893, M47.896, M47.897, M47.891, M47.898, M47.899, M47.894, M47.895, M47.81, M47.813, M47.816, M47.817, M47.811,M47.812, M47.818, M47.819, M47.814, M47.815, M47.1, M47.12, M47.13, M47.16, M4711,

	M47.11, M47.10, M47.14, M47.15, M47.2, M47.22, M47.23, M47.26, M47.27, M47.21, M47.28, M47.20, M47.24, M47.25, M47.9, M49, M49.8
**Infection**	M46.2, M46.22, M46.23, M46.26, M46.27, M46.28, M46.20, M46.24, M46.25, M46.21, M54.9, M54.5, M54.2,M54.50,M54.50, M54, M54.59, M54.51, M54.4, M54.4, M54.42, M54.41, M54.40, M54.8, M54.81, M54.81, M54.89, M54.6, M54.0, M54.03, M54.06, M54.07, M54.01, M54.08, M54.00, M54.04, M54.05, M54.09, M54.1, M54.12, M54.13, M54.16,M54.17,M54.11, M54.18, M54.10, M54.14, M54.15, M54.3, M54.32, M54.31, M54.30
**Symptom/ Syndrome**	G12, G12.0, G12.1, G12.2, G12.21, G12.8, G12.9, G12.20, G12.29, G12.23, G12.22, G82, G82.2, G82.1, G82.22, G82.20, G82.51 G82.5, G82.51, G82.52, G82.53, G82.54, G82.50, G83, G83.4, G83.0, G83.5, G83.1, G83.12, G83.14, G83.11, G83.10, G83.2, G83.22, G83.24, G83.21, G83.23, G83.20, G83.3, G83.32, G83.34, G83.31, G83.33, G83.30, G83.8 , G83.82, G83.81, G83.89, G83.83, G83.84, G83.9, G83.8<br><br>M49.82, M49.83, M49.86, M49.87, M49.81, M49.80, M49.84, M49.85, M50, M50.0, M50.03, M50.01, M50.02, M50.021, M50.022, M50.023, M50.020, M50.00, M50.1, M50,13, M50.11, M50.12, M50.9, M50.121, M50.122, M50.123, M50.120, M50.10, M50.9, M50.93, M50.91, M50.92, M50.920, M50.90, M50.3, M50.33, M50.31,M50.321, M50.322, M50.323, M50.320, M50.30,M50.8, M50.83, M50.81, M50.82, M50.821, M50.822, M50.80, M50.2, M50.23, M50.21, M50.22, M50.20, M51, M51.3, M51.36, M51.37, M51.34, M51.35, M51.2, M51.26, M51.27, M51.24, M51.25, M51.4, M51.46, M51.47, M51.44, M51.45, M51.0, M51.06, M51.04, M51.05, M51.8, M51.86, M51.87, M51.84, M51.85, M51.1, M51.16, M51.17, M51.14, M51.15, M51.9,M53, M53.1, M53.0, M53.9, M53.8, M53.82, M53.83, M53.86, M53.87, M53.81, M53.88, M53.80, M53.84, M53.85, M53.3, M53.2, M53.2x<br><br>S34.12, S34.121, S34.121A, S34.121S, S34.121D, S34.122, S34.122A, S34.122S, S34.122D, S34.123, S34.123A, S34.123A, S34.123S, S34.124, S34.124x, S34.125, S34.125x, S34.129, S34.129x, S34.131, S34.131x, S34.132, S34.132x, S34.139, S34.139x, S34.10,S34.101, S34.101x, S34.102, S34.102A, S34.102S, S34.102D, S34.103, S34.103x, S34.104x,, S34.105, S34.105S, S34.105x<br><br>S35, S35XXx, S33.8, S33.8.XXx, S33.6, S33.6XXx, S33.9, S33.9XXx
**Trauma**	G95, G95.9, G95.2, G95, 29, G95.20, G95.8, G95.81, G95.89, G95.0, G95.1, G95.11, G95.19, G95.9<br><br>S32, S32.4, S32.46, S32.462, S32.462x, S32.461 S32.461x, S32.463, S32.463x, S32.465, S32.465x, S32.464, S32.464x, S32.464D, S32.466, S32.466x, S32.48, S32.482, S32.482x, S32.481, S32.481x, S32.483, S32.483x, S32.485, S32.485x, S32.484, S32.484x, S32.4826, S32.486x, S32.43, S32.43V2, S32.432, S32.432B, S32.432S, S32.432G, S32.432K, S32.432D, S32.431, S32.431x S32.435, S32.435x, S32.434, S32.434A, S32.434B, S32.434S, S32.434G, S32.434S, S32.434K, S32.434D, S32.436, S32.43 S32.436A, S32.436A, S32.436B, S32.436S, S32.436G, S32.436S, S32.436D, S32.41, S32.412, S32.412A, S32.412B, S32.412S, S32.412G, S32.412K, S32.412D, S32.415, S32.415A, S32.415A, S32.415B, S32.415S, S32.415G, S32.415K, S32.415D, S32.411, S32.411, S32.411A, S32.411,B, S32.411S, S32.411G, S32.411K, S32.411D, S32.413, S32.413A, S32.41AB, S32.413S, S32.413G, S32.413K, S32.413D S32.415, S32.415A, S32.415B, S32.415S, S32.415S, S32.415G, S32.415K, S32.415D, S32.414,

	S32.414A, S32.41AB, S32.414S, S32.414G, S32.414K, S32.414D, S32.416, S32.416A, S32.416B, S32.416S, S32.416G, S32.416K, S32.416D, S32.44, S32.444A, S32.444B, S32.444S, S32.444AG, S32.444K, S32.444D, S32.4442, S32.442A, S32.442B, S32.442S, S32.442G, S32.4442K, S32.442D, S32.441, S32.441A, S32.441B, S32.441S, S32.4441G, S32.441K, S32.441D, S32.443, S32.443A, S32.443B, S32.443S, S32.443G, S32.443K, S32.443D, S32.445, S32.4445A, S32.445B, S32.445S, S32.445G, S32.445K, S32.445D, S32.4444, S32.444A, S32.444B, S32.444S, S32.444G, S32.444K, S32.444D, S32.446, S32.446A, S32.446B, S32.446S, S32.446G, S32.446K, S32.446D, S32.47, S32.47A, S32.47B, S32.47S, S32.47G, S32.47K, S32.47D, S32.471, S32.471A, S32.471B, S32.471S, S32.471G, S32.471K, S32.471D, S32.473, S32.473A, S32.473B, S32.473S, S32.473G, S32.473K, S32.473D S32.475, S32.475A, S32.475B, S32.475S, S32.475G, S32.475K, S32.475D, S32.474, S32.474A, S32.474B, S32.474S, S32.474G, S32.474K, S32.474D, S32.476, S32.476A, S32.476B, S32.476S, S32.476G, S32.476K, S32.476D, S32.42, S32.42A, S32.422, S32.422A, S32.422B, S32.422S, S32.422G, S32.422K, S32.422D, S32.421, S32.421A, S32.421B, S32.421S, S32.421G, S32.421K, S32.421D, S32.423, S32.423A, S32.423B, S32.423S, S32.423G, S32.423K, S32.423D, S32.425, S32.425A, S32.425B, S32.425S, S32.425G, S32.425K, S32.425D, S32.424, S32.424A, S32.424B, S32.424S, S32.424G, S32.424K, S32.424D, S32.426, S32.426A, S32.426B, S32.426S, S32.426G, S32.426K, S32.426D, S32.49, S32.4292, S32.4292A, S32.4292B, S32.4292S, S32.4292G, S32.4292K, S32.4292D, S32.4291, S32.4291A, S32.4291S, S32.4291B, S32.4291G, S32.4291K, S32.4291D, S32.4299, S32.4299A, S32.499B, S32.499S, S32.499G, S32.499K, S32.499D, S32.425, S32.452, S32.452A, S32.452B, S32.452S, S32.452G, S32.452K, S32.452D, S32.451, S32.451A, S32.451B, S32.451S, S32.451G, S32.451K, S32.451D, S32.453, S32.453A, S32.453B, S32.453S, S32.453G, S32.453K, S32.453D, S32.455, S32.455A, S32.455B, S32.455S, S32.455G, S32.455K, S32.455D, S32.454, S32.454A, S32.454B, S32.454S, S32.454G, S32.454K, S32.454D, S32.456, S32.456A, S32.456B, S32.456S, S32.456G, S32.456K, S32.456D, S32.40, S32.402, S32.402A, S32.402B, S32.402S, S32.402G, S32.402K, S32.402D, S32.40, S32.401, S32.401A, S32.401B, S32.401S, S32.401G, S32.401K, S32.401D S32.409, S32.409A, S32.409B, S32.409S, S32.409G, S32.409K, S32.409D, S32.2, S32.2XXA, S32.2XXB, S32.2XXS, S32.2XXG, S32.2XXK, S32.2XXD, S32.3, S32.312, S32.312A, S32.312B, S32.312S, S32.312G, S32.312K, S32.312D, S32.311, S32.311A, S32.311B, S32.311S, S32.311G, S32.311K, S32.311D, S32.313, S32.313A, S32.313B, S32.313S, S32.313G, S32.313K,. S32.313D, S32.315, S32.315A, S32.315B, S32.315S, S32.315G, S32.315K, S32.315D, S32.314, S32.314A, S32.314B, S32.314S, S32.314G, S32.314K, S32.314D, S32.316, S32.316A, S32.316B, S32.316S, S32.316G, S32.316K, S32.316D, S32.39, S32.392, S32.392A, S32.392B, S32.392S, S32.392G, S32.392K, S32.392D, S32.399, S32.399A, S32.399B, S32.399S, S32.399G, S32.399K, S32.392D, S32.30, S32.302, S32.302A, S32.302B, S32.302S, S32.302G, S32.302K, S32.302D, S32.301, S32.301A, S32.301B, S32.301S, S32.301G, S32.301K, S32.301D, S32.309, S32.309A, S32.309B, S32.309S, S32.309G, S32.309K, S32.309D, S32.6, S32.61, S32.612, S32.612A, S32.612B, S32.612S, S32.612G, S32.612K, S32.612D, S32.611, S32.612A, S32.611B, S32.611S, S32.611G, S32.611K, S32.611D, S32.613, S32.613A, S32.613B, S32.613S, S32.613G, S32.613K, S32.613D, S32.614, S32.614A, S32.614B, S32.614S,. S32.614G, S32.614K, S32.614D, S32.616, S32.616A, S32.616B, S32.616S, S32.616G, S32.616K, S32.616D, S32.69, S32.692, S32.692A, S32.692B, S32.692S, S32.692G, S32.692K, S32.692D, S32.691, S32.691A, S32.691B, S32.691S, S32.691G, S32.691K, S32.691D, S32.699, S32.699A, S32.699B, S32.699S, S32.699G, S32.699K, S32.699D, S32.60, S32.602, S32.602A, S32.602B, S32.602S, S32.602G, S32.602K, S32.602D, S32.0, S32.05, S32.058, S32.058A, S32.058B, S32.058S, S32.058G, S32.058K, S32.058D, S32.051, S32.051A, S32.051B, S32.051S, S32.051G, S32.051K, S32.051D, S32.059, S32.059A, S32.059B, S32.059S, S32.059G, S32.059K, S32.059D, S32.052, S32.052A, S32.052B, S32.052S, S32.052G, S32.052K, S32.052D, S32.059, S32.059A, S32.059B, S32.059S. S32.059G, S32.059K, S32.059D S32.052, S32.052A, S32.052B, S32.052S, S32.052G, S32.052K, S32.052D, S32.050, S32.050A, S32.050B, S32.050S, S32.050G, S32.050K, S32.050D, S32.01, S32.018, S32.018A, S32.018B,

	S32.018S, S32.018G, S32.018K, S32.018D, S32.011, S32.011A,S32.000A,,S32.000D,S32.000G, S32.001A,S32.001D, S32.001S,S32.009A, S32.009D,S32.009K, S32.009S, S32.020A, S32.011B, S32.011S, S32.011G, S32.011K, S32.011D, S32.019, S32.019A, S32.019B., S32.019S, S32.019G, S32.019K, S32.019D S32.012, S32.012A, S32.012B, S32.012S, S32.012G, S32.012K, S32.012D, S32.010, S32.010A, S32.010B, S32.010S, S32.010G, S32.010K, S32.010D, S32.1, S32.19, S32.19XA, S32.19XB, S32.19XS, S32.19XG, S32.19XK, S32.19XD, S32.14, S32.14XA, S32.14XB, S32.14XS, S32.14XG, S32.14XK, S32.14XD, S32.15, S32.15XA, S32.15XB S32.15XS, S32.15XG, S32.15XK, S32.15XD, S32.16, S32.16XA, S32.16XB, S32.16XS, S32.16XD, S32.16XK, S32.16XG, S32.17, S32.17XA, S32.17XB, S32.17XK, S32.17XS, S32.17XG, S32.17XD, S32.10, S32.10XA, S32.10XB, S32.10XS, S32.10X**G,** S32.10X**K,** S32.10X**D,** S32.11, S32.11XA, S32.11XS, S32.11XB, S32.11XG, S32.11XK, S32.11XD, S32.111, S32.111A, S32.111B, S32.111S, S32.111G, S32.111D, S32.111K, S32.110, S32.110A, S32.110B, S32.110S, S32.110G, S32.110K, S32.110D, S32.112, S32.112A, S32.112B, S32.112S, S32.112G, S32.112K, S32.112D, S32.121, S32.121A, S32.121B, S32.121S, S32.121G, S32.121K, S32.121D, S32.120, S32.120A, S32.120B, S32.120S, S32.120G, S32.120K, S32.120D. S32.122, S32.122A, S32.202D,S32.020G, S32.020S,S32.021A,S32.021D,S32.028A,S32.028D,S32.029A,S32.030A,S32.030S,S32.039D,S32.475A,S32.82XA,S32.9XXA,S32.9XXD,S32.9XXS, S32.122B, S32.122S, S32.122G, S32.122K, S32.122D, S32.129, S32.129A, S32.129B, S32.129B, S32.129S, S32.129G, S32.129K, S32.129D, S32.04, S32.048, S32.048A, S32.048B, S32.048S, S32.048G, S32.048K, S32.048D, S32.041, S32.041A, S32.041B, S32.041S, S32.041G, S32.041K, S32.041D, S32.049, S32.049A, S32.049B, S32.049S, S32.049G, S32.049K, S32.049D, S32.042, S32.042A, S32.042B, S32.042S, S32.042G, S32.042K, S32.042D, S32.040, S32.040A, S32.040B, S32.040S, S32.040G, S32.040K, S32.040D, S32.8, S32.89, S32.89X, S32.89XA, S32.89XB, S32.89XS, S32.89XG, S32.89XK, S32.89XD, S32.81, S32.810, S32.810A, S32.810B, S32.810S, S32.810G, S32.810K, S32.810D, S32.811, S32.811A, S32.811B, S32.811S, S32.811G, S32.811K, S32.811D, S32.82, S32.82A, S32.82B, S32.82S, S32.82G, S32.82K, S32.82D, S32.5, S32.51, S32.512, S32.512A, S32.512B, S32.512S, S32.512G, S32.512K, S32.512D, S32.511, S32.511A, S32.511B, S32.511S, S32.511G, S32.511K, S32.511D, S32.519, S32.519A, S32.519B, S32.519S, S32.519G, S32.519K, S32.519D, S32.59, S32.592, S32.592A, S32.592B, S32.592S, S32.592K, S32.592G, S32.592D, S32.591, S32.591A, S32.591B, S32.591S, S32.591G, S32.591K, S32.591D, S32.599, S32.599A, S32.599B, S32.599S, S32.599G, S32.599K, S32.599D, S32.50, S32.502, S32.502A, S32.502B, S32.502S, S32.502G, S32.502K, S32.502D, S32.501, S32.501A, S32.501B, S32.501S, S32.501G, S32.501K, S32.501D, S32.509 S32.509A, S32.509B, S32.509S, S32.509G, S32.509K, S32.509D, S32.13, S32.131, S32.131A, S32.131B, S32.131S, S32.131G, S32.131K, S32.131D, S32.130, S32.130A, S32.130B, S32.130S, S32.130G, S32.130K, S32.130D, S32.132, S32.132A, S32.132B, S32.132S, S32.132G, S32.132K, S32.132D, S32.139, S32.139A, S32.139B, S32.139S, S32.139G, S32.139K, S32.139D<br><br>S33, S33.3, S33.39, S33.39XA, S33.39XS, S33.39XD, S33.0, S33.0XA, S33.0XS, S33.0XD, S33.2, S33.2XXA, S33.2XXS, S33.2XXD, S33.1, S33.11, S33.111, S33.111A, S33.111S, S33.111D, S33.110, S33.1110A, S33.110S, S33.110D, S33.12, S33.121, S33.121A, S33.1211S, S33.121D, S33.120, S33.120A, S33.120S, S33.120D, S33.13, S33.131, S33.131S, S33.131A, S33.131D, S33.130, S33.130A, S33.130S, S33.130D, S33.14, S33.141, S33.141A, S33.141S, S33.141D, S33.140, S33.140A, S33.140S, S33.140D, S33.10, S33.101, S33.101A, S33.101S, S33.101D, S33.100, S33.100A, S33.100S, S33.100D, S33.0, S33.0XXA, S33.0XXS, S33.0XXD, S33.4, S33.4XXA, S33.4XXS, S33.4XXD,S33.5XXA,S33.5XXD

	S34.0, S34.01, S34.01XA, S34.01XS, S34.01XD, S34.02, S34.02XA, S34.02XS, S34.02XD ,S39.82, S39.82XA, S39.82XS, S39.82XD, S39.092, S39.092A, S39.092S, S39.092D, S39.012, S39.012A, S39.012S, S39.012D, S39.92XA, S39.91XA, S39.011, S34.3, S34.3XXx, S34.5, S34.5XXx, S34.4, S34.4XXx, S34.2, S34.21, S34.21Xx, S34.22, S34.22Xx, S34.8, S34.8XXA, S34.XXS, S34.8XXD, S34.6, S34.6XXA, S34.6XXx, S34.9, S34.9XXx, S34.1, S34.11, S34.111, S34.111x, S34.112, S34.112x, S34.113, S34.113x, S34.114, S34.114x, S34.115, S34.115x, S34.119, S34.119Ax
